# Management of Sunken Cheeks With Magnet-Retained Cheek Plumpers

**DOI:** 10.7759/cureus.36161

**Published:** 2023-03-14

**Authors:** Ramesh Kunusoth, Himaja Swayampakula, Shreya Colvenkar, Radhika D, Aditya Mohan Alwala

**Affiliations:** 1 Department of Oral and Maxillofacial Surgery, MNR Dental College and Hospital, Sangareddy, IND; 2 Department of Prosthodontics, MNR Dental College and Hospital, Sangareddy, IND; 3 Department of Prosthodontics, Kamineni Institute of Dental Sciences, Narketpally, IND

**Keywords:** aging, cheeks, aesthetic, magnet, complete denture, cheek plumper

## Abstract

The loss of teeth, associated with sunken lips and cheeks, has a debilitating psychological effect on a person. It is essential for clinicians to incorporate facial esthetics into their treatment plan for complete denture patients in order to improve the patients’ confidence and quality of life. Cheek plumpers ensure adequate support to facial muscles, which, in turn, minimizes the appearance of wrinkles, lines, and sagging over time. This case report describes the fabrication of detachable cheek plumpers using magnets to enhance the facial esthetics of a completely edentulous patient. Magnet-retained cheek plumpers, being small and lightweight, allow ease of placement and cleaning without the addition of weight to the prosthesis.

## Introduction

Facial esthetics is increasingly seen as an important factor for the overall health and well-being of individuals [1]. Natural dentition plays a vital role in providing support to the facial musculature, which is crucial for structural stability and esthetics of the facial features, including the lips and cheeks. When these elements are disturbed, it becomes difficult to achieve a proper and natural-looking external form of the face [2].

With aging, the processes and functions of our bodies change. One of the most affected parts is the midface area, considered to be the most prominent facial region. Aging comes with changes in not only how these areas look but also in how they function. This causes several age-related ailments such as wrinkles, sagging cheeks and lips, and volume loss [3].

Cheek plumper, also known as the cheek lifting appliance, provides an effective way of lifting and supporting the cheeks. It is a type of prosthesis that acts as a foundation for the facial structure, providing support and structure to the face [4-8]. It is a safe and effective option for those seeking a more youthful, toned look without surgery or other invasive procedures. It can improve self-confidence in people who have sagging or hollow-looking cheeks or lack facial definition due to aging. Additionally, it can be an effective solution when aiming to correct asymmetry on both sides of the face [8]. Cheek plumpers can be classified into detachable [4] and undetachable types [5-8]. In the undetachable type, a cheek plumper is built within the denture. Its disadvantages include muscle fatigue, increased weight, which compromises retention of the denture, and limitation of use in microstomia patients [9].

The detachable cheek plumpers provide advantages over the conventional, undetachable ones. The ability to easily attach and remove them offers convenience, control, and comfort [5]. They prevent muscle fatigue caused by long-term wear and are also easy to clean. Attachments such as press buttons [5], double die pins [6], and customized attachments [7] can be used. Customized attachments necessitate more time and additional laboratory steps [7]. Although press buttons are inexpensive and fit snugly, they are not corrosion resistant [5]. When using a double die pin, the patient must exercise caution because the pin can cause trauma to the mucosa if not inserted properly [6]. Compared to other known methods, the magnet-retained cheek plumper allows for ease of cleaning and placement and automatic reseating due to strong magnetic force. In addition, the denture flange can easily accommodate smaller magnets.

This case report describes the fabrication of detachable cheek plumpers using magnets to enhance the facial esthetics of a completely edentulous patient.

## Case presentation

**Figure 1:**
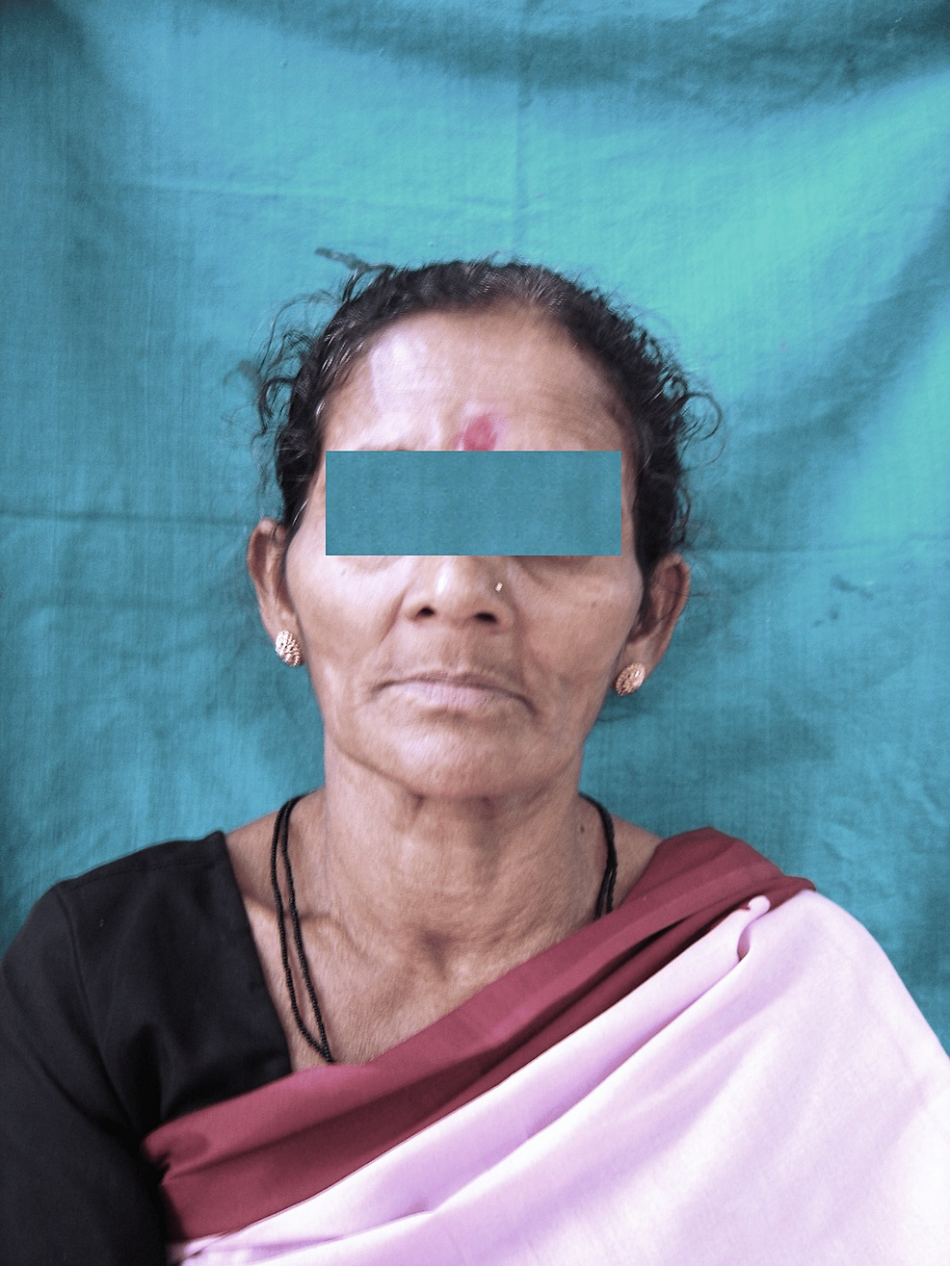
Pre-treatment photograph

A 65-year-old female patient with no history of trauma presented to the department of prosthodontics for the replacement of missing teeth that had resulted in the inability to eat. The patient also complained of sunken cheeks (Figure 1).

The history of the patient revealed that she lost the teeth gradually over a period of four years after they became mobile. She was edentulous for a period of two years. On intra-oral examination, the patient was found to have moderately resorbed maxillary and mandibular edentulous ridges. Extraoral examination revealed sunken cheeks and unsupported oral musculature. The patient’s straightforward request was to improve facial esthetics while replacing the missing teeth. Various treatment options were discussed with the patient, subsequent to which she opted for a removable complete denture with detachable cheek plumpers.

**Figure 2:**
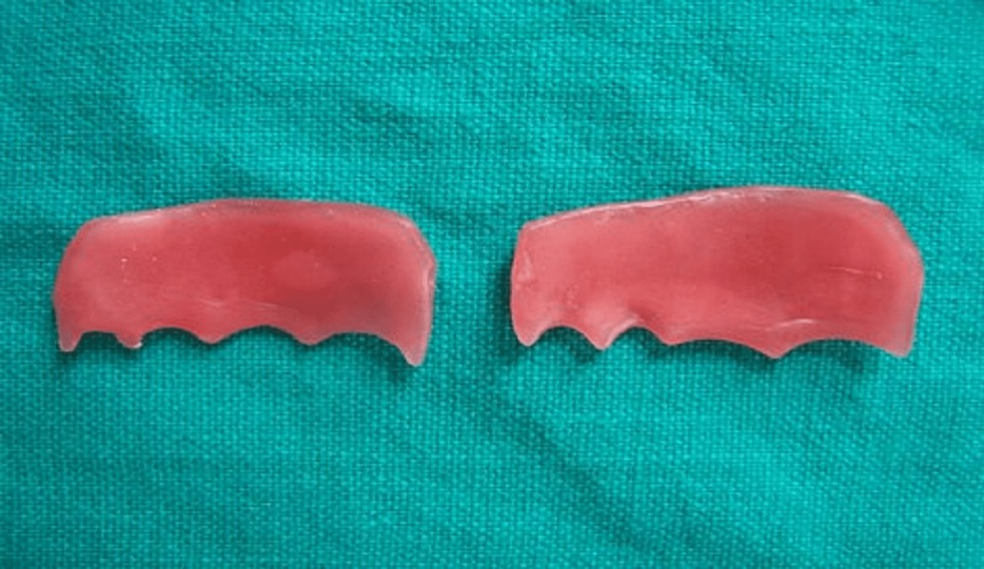
Cheek plumper fabricated in wax

All steps in complete denture fabrication were completed to the try-in stage. At the try-in stage, cheek plumpers were made in wax as separate portions on the buccal surface of the complete trial denture (Figure 2).

**Figure 3:**
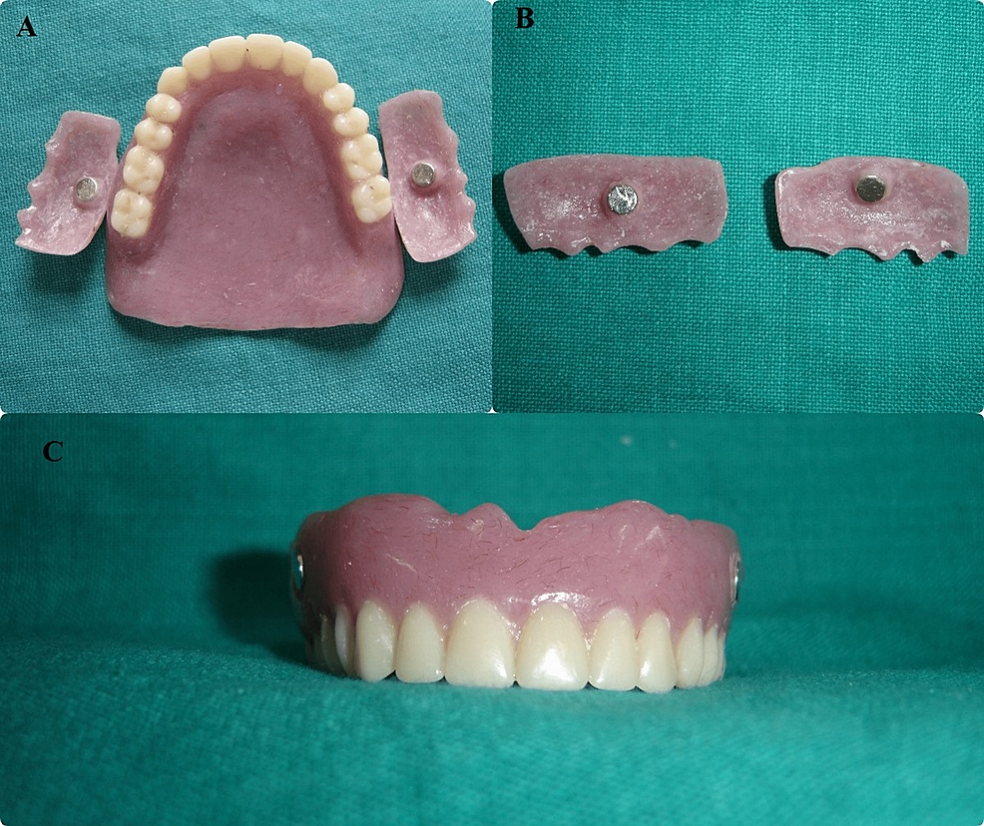
A - Cheek plumpers with denture. B - Magnet in cheek plumper C-magnet in denture

The cheek plumpers were superficially attached to the buccal surfaces on the right and left sides and tried in the mouth to determine the amount of desired cheek support appropriate for comfort, function, and esthetics. Once this was determined, they were again separated from the complete denture. The dentures and cheek plumpers were processed separately. Button magnets (Rare Earth Magnet, Permag Products Pvt. Ltd., Pune, India) that were encased in steel casing were then placed in each of the hollowed buccal extensions using self-cure acrylic (Figure 3).

**Figure 4:**
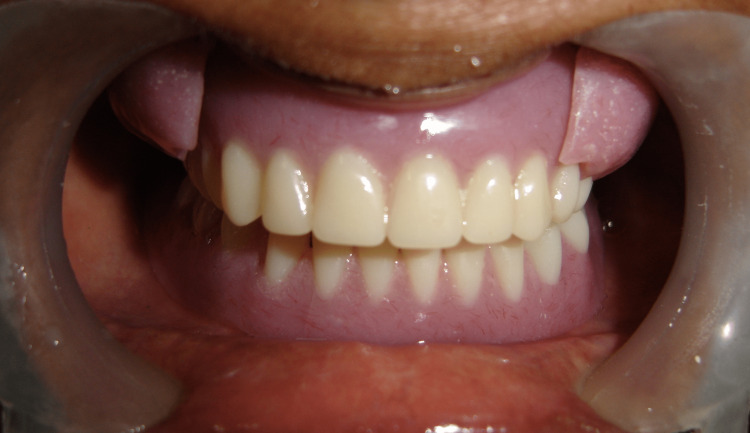
Intraoral view of denture with cheek plumper

Corresponding to this buccal extension, hollowed cavities were made on the buccal surface of the denture on the right and left sides approximately in the molar region. Magnets were then placed in these cavities using self-cure acrylic resin and care was taken to align the poles properly. The cheek plumpers snapped into position as a result of strong, attractive forces between the magnet in the polished buccal surface of the denture and the intaglio surface of the buccal extension. During denture delivery, the patient was educated on how to insert the denture and cheek plumpers (Figure 4).

**Figure 5:**
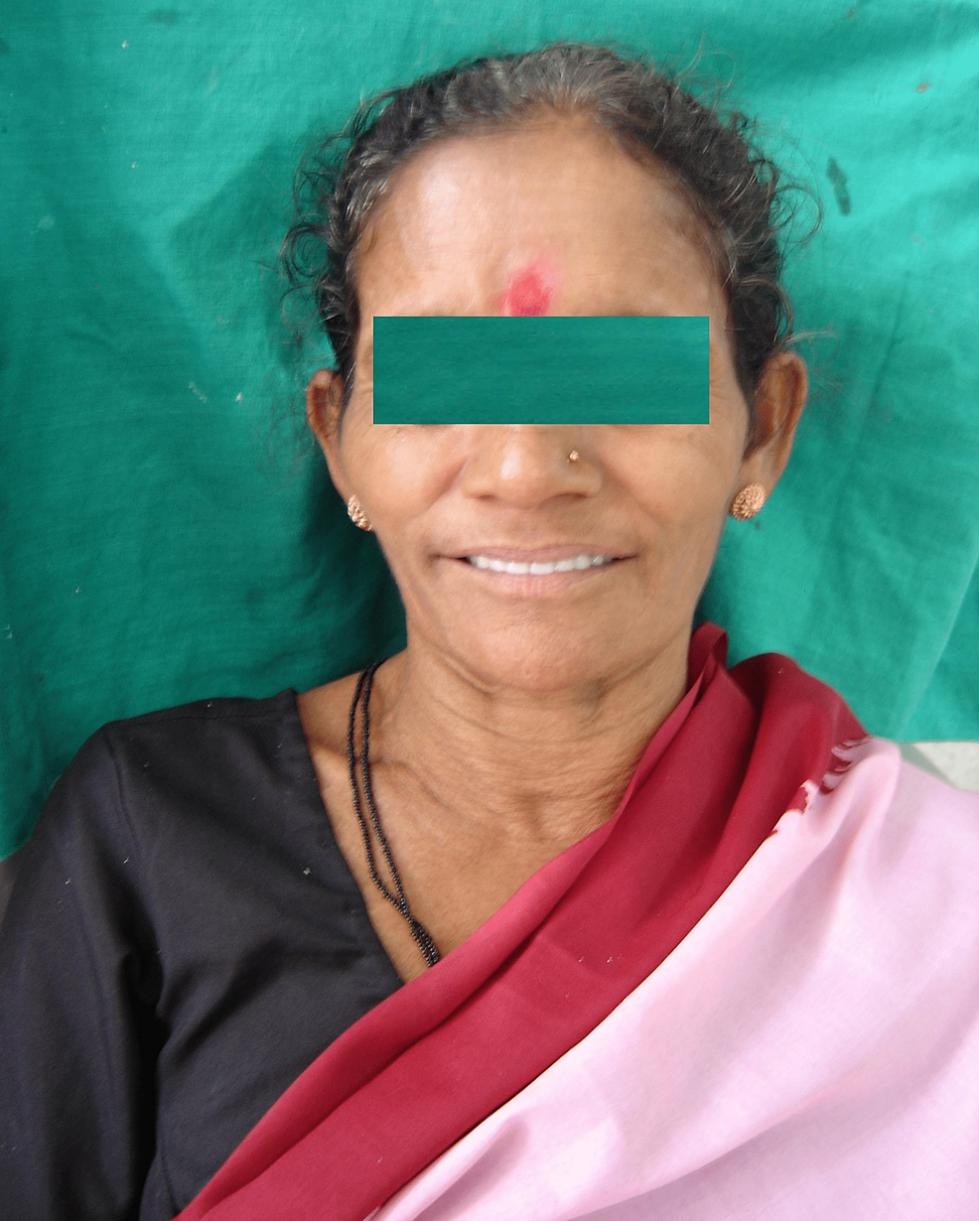
Post-treatment photograph

The patient was asked to insert the complete denture followed by a cheek plumper on either side (Figure 5).

Periodic follow-up revealed that the patient adjusted very well to the prosthesis.

## Discussion

Loss of teeth, associated with sunken lips and cheeks, has a debilitating psychological effect on a person [1]. Proper support to the facial muscles is required to minimize the appearance of wrinkles, lines, and sagging over time. In the absence of teeth, adequate support to the musculature can be achieved by using cheek plumpers [4-8]. The undetachable cheek plumper increases the size and weight of dentures, thereby compromising the retention of the dentures. In addition, excessive mediolateral width of the dentures in the region of the cheek plumper prevents insertion of the dentures for patients with microstomia [9].

Detachable cheek plumpers provide multiple advantages such as easy insertion and removal, reduced height, prevention of muscle fatigue, and easier cleaning. The patient can wear the dentures even without cheek plumpers if muscle fatigue sets in. Various attachments such as press buttons [5], double die pin [6], and customized attachments [7] can be used. Customized attachments require more time and additional laboratory steps for fabrication [7]. Although press buttons are cheap and fit snugly, they are not resistant to corrosion [5]. The patient needs to be careful while using double die pins as the pins can hurt and cause trauma to the mucosa if not inserted properly [6].

Magnet-retained cheek plumpers can be discretely and easily worn due to their lightweight [10]. The plumpers can be removed from the mouth during eating and when experiencing excessive muscle fatigue. It allows ease of cleaning and placement in the mouth without the addition of weight to the prosthesis. According to a study by Bondemark L and his colleagues, static magnetic fields do not appear to result in any changes to the human dental pulp or gingival tissues adjacent to the magnets [11]. Also, in a clinical, histological, and immunohistochemical study, Bondemark L et al. found no adverse, long-term effects on the human buccal mucosa that had been in contact with acrylic-coated neodymium iron boron magnets and subjected to the static magnetic field [12].

Limitation of magnet retained cheek plumpers are magnets that cannot be used on patients who are allergic to metal. Patients should be informed that the magnetic field used in magnetic resonance imaging (MRI) tests causes damage to the magnetic assembly. For MRI tests, patients must remove their dentures. The magnetic assembly should be kept away from temperatures exceeding 150°C.

Reconstructive plastic surgery, injections of botulinum toxin, and tightening procedures such as laser resurfacing are all known to be effective methods for correction [13]. However, elderly patients with systemic disease prefer easy, non-invasive procedures due to cost and time factors. Hence, magnet-retained cheek plumpers are an easy go-to option for many people looking to improve their appearance and facial contours.

## Conclusions

The functioning of various muscles in the face is hindered when the lips and cheeks are not supported. This leads to wrinkles and a sagging appearance of the lips and cheeks. This case report presents a successful management of sunken cheeks using detachable magnet-retained cheek plumpers. Magnet-retained cheek plumpers, being small and lightweight, allow ease of placement and cleaning in the mouth without the addition of weight to the prosthesis.
